# Association between economic status and body mass index among adolescents: a community-based cross-sectional study in Japan

**DOI:** 10.1186/s40608-016-0127-z

**Published:** 2016-11-10

**Authors:** Akiko Mizuta, Takeo Fujiwara, Toshiyuki Ojima

**Affiliations:** 1 Department of Community Health Nursing, Hamamatsu University School of Medicine, 1-20-1 Handayama, Higashi-ku, Hamamatsu, Shizuoka 431-3192 Japan; 2 Department of Global Health Promotion, Tokyo Medical and Dental University (TMDU), 1-5-45 Yushima, Bunkyo-ku, Tokyo, 113-8519 Japan; 3 Department of Community Health and Preventive Medicine, Hamamatsu University School of Medicine, 1-20-1 Handayama, Higashi-ku, Hamamatsu, Shizuoka 431-3192 Japan

**Keywords:** BMI, BMI *z*-score, Economic status, Adolescent, Breakfast skipping

## Abstract

**Background:**

Childhood overweight and obesity is a growing health challenge in Japan and might be associated with childhood poverty.

We aimed to investigate the association between low economic status and body mass index (BMI) and to reveal the mediators of this association among junior high school students in Japan.

**Methods:**

Junior high school students (*N* = 2968) from two cities in Shizuoka, Japan, were surveyed. Questionnaires assessed subjective economic status, weight, and height. Economic status was categorized into low and non-low, and BMI z-scores were calculated using the WHO Growth Reference. Multivariate regression analyses were conducted to determine the association between economic status and BMI z-scores, adjusted for covariates and stratified by gender.

**Results:**

Among girls, low economic status was significantly positively associated with BMI z-scores in the crude model (coefficient: 0.35; *p* = 0.001). In a model adjusted for breakfast skipping, the coefficient of economic status decreased by 28.57 % but remained significant (coefficient: 0.25; *p* = 0.017). In the final model adjusted for other possible covariates, low economic status remained significantly positively associated with BMI z-score (coefficient: 0.22; *p* = 0.044). The same association was not found for boys.

**Conclusions:**

Low economic status was positively associated with higher BMI among girls in junior high school in Japan, but this was not true for boys. Childhood poverty might be associated with overweight and obesity among adolescent girls in Japan. Health policies at junior high schools to discourage breakfast skipping might be effective for countering the association between childhood poverty and overweight in adolescent girls.

**Electronic supplementary material:**

The online version of this article (doi:10.1186/s40608-016-0127-z) contains supplementary material, which is available to authorized users.

## Background

Childhood poverty is a risk factor for disease in adulthood and can have a significant impact on long-term health. In Japan, the relative child poverty rate was 15.7 % in 2010, meaning that one in six children grows up in poverty across the country [1]. Previous studies have reported on the association between socioeconomic status and childhood overweight and obesity [2, 3], and the relationships of childhood overweight with cardiovascular risk factors [4], cardiovascular disease [5], asthma [6], depression [7], and premature mortality [8, 9]. Moreover, childhood overweight and obesity have an adverse effect on socioeconomic status in young adulthood, such as lower educational attainment and income [10].

The rates of overweight and obesity among children are increasing globally, and the Asia-Pacific region is no exception [11–13]. The prevalence of obesity among Japanese schoolchildren in the 7th, 8th, and 9th grades was 9.13 %, 8.04 %, and 7.55 %, respectively, in 2015 [14]. Although these rates are relatively low compared with those in other developed countries [15], they have increased since 1977 (7th grade, 6.64 %; 8th grade, 5.63 %; and 9th grade. 4.91 %) [14]. In Japan, risk of childhood obesity might be related to irregular intake of breakfast [16, 17]. Although these risk factors are likely to be associated with childhood poverty [18], few studies have investigated the association between socioeconomic status and childhood overweight and obesity in Japan.

The aim of this study was to clarify the relationship between economic status and body mass index (BMI) among junior high school students in Japan.

## Methods

### Participants

Students were recruited from all three grades (7th, 8th, and 9th) of all eight public junior high schools located in Kikugawa City and Kosai City, both of which are suburban areas within Shizuoka Prefecture, located in central Japan (*N* = 2968). The 7th, 8th, and 9th grades in Japan are generally for students aged 13, 14, and 15 years, respectively. In the Japanese education system, junior high school is a lower-secondary school that comes after elementary school and before senior high school. The survey was conducted between December 2012 and January 2013. Homeroom teachers guided students in filling out the surveys during class time, and completed questionnaires were collected in sealed envelopes. Completion of the anonymous self-assessment questionnaire was considered consent to participate in the study. Completed questionnaires were then collected and placed in sealed envelopes individually. Questionnaire of students who rejected this survey were also collected remains blank, in the same way as other students. (Additional file 1).

### Ethics statement

Written explanations of the study were provided to students, parents, and guardians, as well as homeroom teachers and principals. Those who did not wish to participate in the study could decline to respond. Written informed consent was assumed by voluntary response of the anonymous questionnaire. In Japan, according to ethical guidelines for epidemiological research, written informed consent is not necessary for observational research that does not collect human biological specimens, such as blood or urine.

### Measurement

Weight and height were self-reported in the questionnaire in kilograms and centimeters respectively, and recorded to the first decimal place. Previous studies in Japan have shown that self-reported child weight and height is highly accurate [19]. BMI (kg/m^2^) was calculated as weight (kg) over height squared (m^2^). To adjust for child age and gender, BMI *z*-scores were calculated based on WHO standards [20].

Because it would be difficult to obtain exact figures of students’ annual household income, we assessed economic status using a subjective measurement of economic status as follows: “How do you rate your household living-conditions as financial situation?” This item was scored on a 5-point Likert scale: (1) “difficult,” (2) “somewhat difficult,” (3) “normal,” (4) “somewhat comfortable,” and (5) “comfortable.” This subjective measurement of economic status was used in the 2010 Comprehensive Survey of Living Conditions conducted by the Japanese Ministry of Health, Labour and Welfare [21] and validated by comparing the question with annual household income reported in another study [22]. To focus on childhood poverty, we divided economic status into two categories: “low” (difficult: boys 4.28 %, girls 3.35 %) and “non-low” (somewhat difficult: boys 14.88 %, girls 19.06 %; normal: boys 63.56 %, girls 60.72 %; somewhat comfortable: boys 12.92 %, girls 10.33 %; and comfortable: boys 4.36 %, girls 6.53 %).

### Covariates

The questionnaire included questions about sociodemographic factors such as gender, date of birth, grade in school, lifestyle topics including eating, activity, sleeping patterns and smoking status, self-rated health status, family members’ smoking status and, family structure. Breakfast skipping and the number of meals from convenience stores and fast-food outlets (convenience stores and fast-food outlets) were rated if the frequency of skipping breakfast and eating meals from convenience stores or fast-food outlets were ≥ 1 time per week. We also assessed participation in club activities at school (club activities at school) (“physical,” “non-physical,” “both physical and non-physical,” or “no participation”), and participation in physical and non-physical activities outside of school (activities outside of school) with a yes/no. In the Japanese education system, public junior high schools provide non-compulsory club activities that are either physical (e.g., baseball club and soccer club) or non-physical (e.g., watching movies, brass band, and chorus). For sleeping patterns, we assessed the number of times students got up and went to bed each night. Sleeping patterns were then divided by two, using a less than first quintile cut-off (less than 6.5 h per night). Self-rated health status was scored on a 5-point Likert scale of (1) “excellent,” (2) “good,” (3) “fair,” (4) “somewhat poor,” and (5) “poor,” which was then reorganized into two categories of “good” (excellent, good, and fair) and “not good” (somewhat poor, poor). We used a self-rated question for health status obtained from the 2010 Comprehensive Survey of Living Conditions [21], which was correlated with the Birleson depression self-rating scale (DSRS) (boys: Spearman’s *r* = 0.43, *p* < 0.001; girls: Spearman’s *r* = 0.48, *p* < 0.001). Smoking status was assessed using the question “Have you ever smoked before (even if only once)?” with a yes/no response. As for family members who lived together, we divided students into family structure of “living with both of their biological parents” and “those living in a single-parent family or stepfamily. Finally, family members’ smoking status (father, mother, and other) was rated and classified into two categories of “yes” (≥1 person) and “no” (none).

### Statistical analysis

We examined the association between economic status and BMI *z*-scores using linear regression analysis. Univariate regression analysis was conducted in Model 1, and school, grade, and family structure were added to Model 2 as confounding variables. In Models 3, 4, 5, 6, and 7, we added the following possible mediators: family members’ smoking status (Model 3), breakfast skipping (Model 4), activities outside of school (Model 5), sleeping patterns (Model 6), and self-rated health status (Model 7), owing to estimate change of coefficient. Finally, multivariate regression analysis was performed for economic status using explanatory variables and an objective variable of BMI *z*-scores, and was adjusted for all confounders and all possible mediators (Model 8). We used STATA version 13.0 (STATA Corp LP., College Station, TX, USA) for statistical analysis.

## Results

### Participant characteristics

Of the 2968 students recruited for the questionnaire, 106 were absent on the day of distribution. Therefore, questionnaires were distributed to 2862 students. A total of 2302 students completed all questionnaire items and were included in the analysis (valid response rate, 77.56 %). We cannot assess the difference between responders and non-responders as we lacked data on non-responders. Table 1 shows participant characteristics. In our sample, 4.53 % of boys and 3.18 % of girls were underweight (BMI *z*-score < −2 *SD*), 8.04 % of boys and 5.91 % of girls were overweight (BMI *z*-score > +1 *SD*), and 1.20 % of boys and 1.15 % of girls were obese (BMI *z*-score > +2 *SD*). Under low economic status, there was a total of 4.28 % of boys and 3.35 % of girls. Grade in school, economic status, family structure, and family members’ smoking status did not differ by gender. Breakfast skipping, activities outside of school, short sleep duration, and poor self-rated health status were more likely to be found among girls than boys. Convenience stores and fast-food outlets and smoking status were more likely to be found among boys than girls.

**Table 1:** Characteristics of Participants

	Boys (*n* = 1169)		Girls (*n* = 1133)	
	*n* or mean	% or *SD*	*n* or mean	% or *SD*
Family characteristics				
Economic status				
Non-low (comfortable, somewhat comfortable, normal, somewhat difficult)	1119	(95.72)	1095	(96.65)
Low (difficult)	50	(4.28)	38	(3.35)
Family structure				
Both biological parents	964	(82.46)	928	(81.91)
Single parent or stepfamily	205	(17.54)	205	(18.09)
Family members’ smoking status (mother, father, or others)				
None	565	(48.33)	527	(46.51)
Yes (≥1 person)	604	(51.67)	606	(53.49)
Child characteristics				
BMI	18.92	(2.63)	19.19	(2.57)
BMI *z*-score (WHO standard)	−0.17	(0.68)	−0.18	(0.63)
< −2 *SD*	53	(4.53)	36	(3.18)
≥ −2 *SD* and < −1 *SD*	215	(18.39)	220	(19.42)
≥ −1 *SD* and < +1 *SD*	793	(67.84)	797	(70.34)
≥ +1 *SD* and < +2 *SD*	94	(8.04)	67	(5.91)
≥ +2 *SD*	14	(1.20)	13	(1.15)
Grade in school				
7th	396	(33.88)	387	(34.16)
8th	382	(32.68)	361	(31.86)
9th	391	(33.45)	385	(33.98)
Breakfast skipping				
No (eat breakfast daily)	1032	(88.28)	963	(85.00)
Yes (skip breakfast ≥ 1 time per week)	137	(11.72)	170	(15.00)
Convenience stores or fast-food outlets				
< 1 time per week	959	(82.04)	961	(84.82)
≥ 1 time per week	210	(17.96)	172	(15.18)
Club activities at school				
Physical	850	(72.71)	575	(50.75)
Non-physical	77	(6.59)	322	(28.42)
No participation	242	(20.70)	236	(20.83)
Activities outside of school				
Yes	398	(34.05)	466	(41.13)
No	771	(65.95)	667	(58.87)
Sleeping patterns				
≥ 6 h 30 min per day (Q2–Q5)	955	(81.69)	826	(72.90)
< 6 h 30 min per day (Q1)	214	(18.31)	307	(27.10)
Self-rated health status				
Good (excellent, good, fair)	1084	(92.73)	1033	(91.17)
Not good (somewhat poor or poor)	85	(7.27)	100	(8.83)
Smoking status				
No	1,126	(96.32)	1107	(97.71)
Yes	43	(3.68)	26	(2.29)

### Economic status and covariates

Table 2 shows the association between economic status and possible confounders and mediators. For boys, no association was observed between economic status and possible mediators. For girls, breakfast skipping, sleeping patterns, self-rated health status, and smoking status were significantly associated with economic status.

**Table 2:** Association Between Economic Status and Possible Mediators

	Boys					Girls				
	Non-Low		Low		*p*	Non-Low		Low		*p*
	*n*	%	*n*	%		*n*	%	*n*	%	
Family characteristics										
Family structure										
Both biological parents	926	(96.06)	38	(3.94)	0.219	910	(98.06)	18	(1.94)	<0.001
Single parent or stepfamily	193	(94.15)	12	(5.85)		185	(90.24)	20	(9.76)	
Family members’ smoking status (mother, father, or others)										
None	542	(95.93)	23	(4.07)	0.736	514	(97.53)	13	(2.47)	0.122
Yes (≥1 person)	577	(95.53)	27	(4.47)		581	(95.87)	25	(4.13)	
Child characteristics										
Breakfast skipping										
No (eat breakfast daily)	990	(95.93)	42	(4.07)	0.336	944	(98.03)	19	(1.97)	<0.001
Yes (skip breakfast ≥ 1 time per week)	129	(94.16)	8	(5.84)		151	(88.82)	19	(11.18)	
Convenience stores or fast-food outlets										
< 1 time per week	916	(95.52)	43	(4.48)	0.455	929	(96.67)	32	(3.33)	0.915
≥ 1 time per week	203	(96.67)	7	(3.33)		166	(96.51)	6	(3.49)	
Club activities at school										
Physical	819	(96.35)	31	(3.65)	0.065	562	(97.74)	13	(2.26)	0.092
Non-physical	70	(90.91)	7	(9.09)		309	(95.96)	13	(4.04)	
No participation	230	(95.04)	12	(4.96)		224	(94.92)	12	(5.08)	
Activities outside of school										
Yes	378	(94.97)	20	(5.03)	0.364	456	(97.85)	10	(2.15)	0.059
No	741	(96.11)	30	(3.89)		639	(95.80)	28	(4.20)	
Sleeping patterns										
≥ 6 h 30 min (Q2-Q5)	915	(95.81)	40	(4.19)	0.752	805	(97.46)	21	(2.54)	0.013
< 6 h 30 min (Q1)	204	(95.33)	10	(4.67)		290	(94.46)	17	(5.54)	
Self-rated health status										
Good (excellent, good, fair)	1041	(96.03)	43	(3.97)	0.061	1008	(97.58)	25	(2.42)	<0.001
Not good (somewhat poor or poor)	78	(91.76)	7	(8.24)		87	(87.00)	13	(13.00)	
Smoking status										
No	1079	(95.83)	47	(4.17)	0.373	1072	(96.84)	35	(3.16)	0.019
Yes	40	(93.02)	3	(6.98)		23	(88.46)	3	(11.54)	

*Note*. Chi square test

### Economic status and possible mediators related to BMI z-scores

Among boys, low economic status was not associated with BMI *z*-scores (coefficient: −0.07, *p* = 0.460). However, among girls, low economic status was significantly associated with BMI *z*-scores (coefficient: 0.35, *p* = 0.001). In Model 2, this association remained significant among girls (coefficient: 0.32, *p* = 0.002).

Models 3 to 7 show coefficients of low economic status for BMI *z*-scores adjusted for possible mediators. For boys, all models showed no correlation between low economic status and BMI *z*-score. For girls, when Model 4 was adjusted for breakfast skipping, the coefficient of economic status decreased by 28.57 % (coefficient: 0.25, *p* = 0.017). Furthermore, when Model 8 was adjusted for all possible mediators, the coefficient of economic status for BMI *z*-score remained significant for girls (coefficient: 0.21, *p* = 0.044). For simple Bonferroni correction a significance threshold of < 0.006 (0.05/8). At this threshold, economic status remained a significant predictor of obesity in girls. In addition, family structure and sleeping patterns among boys (Table 3) and breakfast skipping and activities outside of school among girls (Table 4) remained significant at this threshold.

**Table 3:** Relationship between economic status and BMI *Z*-Scores of Boys

	Model 1^a^			Model 2^b^ (+ grade, school, family structure)			Model 3 (Model 2 + family members’ smoking status)			Model 4 (Model 2 + breakfast skipping)			Model 5 (Model 2 + activities outside of school)			Model 6 (Model 2 + sleeping patterns)			Model 7 (Model 2 + self-rated health status)			Model 8^c^ (Model 2 + all mediators)		
	*B*	*SE*	*p*	*B*	*SE*	*p*	*B*	*SE*	*p*	*B*	*SE*	*p*	*B*	*SE*	*p*	*B*	*SE*	*p*	*B*	*SE*	*p*	*B*	*SE*	*p*
Family characteristics																								
Economic status																								
Low (ref: non-low)	−0.07	0.10	0.460	−0.10	0.10	0.327	−0.10	0.10	0.326	−0.10	0.10	0.312	−0.10	0.10	0.302	−0.10	0.10	0.318	−0.10	0.10	0.291	−0.11	0.10	0.241
Family structure																								
Single parent or stepfamily (ref: both biological parents)	0.15^**^	0.05	0.005	0.15^**^	0.05	0.005	0.15^**^	0.05	0.005	0.14^**^	0.05	0.009	0.15^**^	0.05	0.004	0.15^**^	0.05	0.004	0.15^**^	0.05	0.005	0.14^**^	0.05	0.006
Family members’ smoking status																								
Yes (ref: none)	0.003	0.04	0.944				0.014	0.04	0.725													0.01	0.04	0.798
Child characteristics																								
Breakfast skipping																								
Yes (ref: no)	0.13^*^	0.06	0.029							0.11	0.06	0.083										0.08	0.06	0.199
Convenience stores or fast-food outlets																								
<1 time per week (ref: ≥ 1 time per week)	−0.01	0.05	0.802																			−0.05	0.05	0.343
Club activities at school																								
Non-physical (ref: physical)	0.11	0.08	0.193																			0.02	0.08	0.804
No participation (ref: physical)	0.03	0.05	0.538																			0.03	0.07	0.665
Activities outside of school																								
No (ref: yes)	−0.06	0.04	0.130										−0.07	0.04	0.078							−0.08	0.04	0.049
Sleeping patterns																								
<6 h 30 min (ref: ≥ 6 h 30 min)	0.19^***^	0.05	<0.001													0.18^***^	0.05	<0.001				0.17^**^	0.05	0.001
Self-rated health status																								
Not good (ref: good)	0.09	0.08	0.217																0.11	0.08	0.151	0.08	0.08	0.326
Smoking status																								
Yes (ref: no)	0.13	0.11	0.226																			0.04	0.11	0.736

*B* = Partial regression coefficient, ^a^univariate regression; ^b^multiple regression adjusted for confounding variables; ^c^multiple regression adjusted for all mediators

^*^ *p* < 0.05; ^**^ *p* < 0.01; ^***^ *p* < 0.001

**Table 4:** Relationship Between Economic Status and BMI *Z*-Scores of Girls

	Model 1^a^			Model 2^b^ (+ grade, school, family structure)			Model 3 (Model 2 + family members’ smoking status)			Model 4 (Model 2 + breakfast skipping)			Model 5 (Model 2 + activities outside of school)			Model 6 (Model 2 + sleeping patterns)			Model 7 (Model 2 + self-rated health status)			Model 8^c^ (Model 2 + all mediators)		
	*B*	*SE*	*p*	*B*	*SE*	*p*	*B*	*SE*	*p*	*B*	*SE*	*p*	*B*	*SE*	*p*	*B*	*SE*	*p*	*B*	*SE*	*p*	*B*	*SE*	*p*
Family characteristics																								
Economic status																								
Low (ref: non-low)	0.35^**^	0.10	0.001	0.32^**^	0.10	0.002	0.31^**^	0.10	0.003	0.25^*^	0.11	0.017	0.31^**^	0.10	0.003	0.32^**^	0.11	0.003	0.30^**^	0.11	0.005	0.21^*^	0.11	0.044
Family structure																								
Single parent or stepfamily (ref: both biological parents)	0.14^**^	0.05	0.005	0.11^*^	0.05	0.021	0.11^*^	0.05	0.021	0.08	0.05	0.094	0.11^*^	0.05	0.026	0.11^*^	0.05	0.022	0.11^*^	0.05	0.023	0.07	0.05	0.139
Family members’ smoking status																								
Yes (ref: none)	0.08^*^	0.04	0.042				0.07^*^	0.04	0.049													0.05	0.04	0.211
Child characteristics																								
Breakfast skipping																								
Yes (ref: no)	0.26^***^	0.05	<0.001							0.22^***^	0.05	<0.001										0.21^***^	0.05	<0.001
Convenience stores or fast-food outlets																								
<1 time per week (ref: ≥ 1 time per week)	−0.03	0.05	0.529																			−0.06	0.05	0.220
Club activities at school																								
Non-physical (ref: physical)	0.07	0.04	0.120																			0.03	0.04	0.448
No participation (ref: physical)	−0.04	0.05	0.379																			−0.05	0.07	0.450
Activities outside of school																								
No (ref: yes)	0.12^**^	0.04	0.001										0.10^*^	0.04	0.010							0.10^*^	0.04	0.012
Sleeping patterns																								
<6 h 30 min (ref: ≥ 6 h 30 min)	0.07	0.04	0.113													0.04	0.04	0.394				0.004	0.04	0.921
Self-rated health status																								
Not good (ref: good)	0.16^*^	0.07	0.015																0.10	0.07	0.130	0.08	0.07	0.250
Smoking status																								
Yes (ref: no)	0.18	0.12	0.150																			0.11	0.12	0.389

*B* = Partial regression coefficient, ^a^univariate regression; ^b^multiple regression adjusted for confounding variables; ^c^multiple regression adjusted for all mediators

^*^ *p* < 0.05; ^**^ *p* < 0.01; ^***^ *p* < 0.001

## Discussion

We found that low economic status was significantly associated with high BMI *z*-score for adolescent girls, independent of family structure or lifestyle factors such as physical activity. Further, we found that breakfast skipping could be a possible mediator of this association. To the best of our knowledge, this population-based study is the first to show an association between childhood poverty and obesity among adolescent Japanese girls.

In the United States, child obesity is associated with low economic status [23], although it is also associated with high economic status [24]. A study of four European countries that used different measures of economic status found no association between parental education and childhood BMI [25]. Thus, differences in race or other possible confounding factors related to culture should be considered when interpreting our findings. Nonetheless, our study adds to the literature by finding an association between childhood poverty and overweight among adolescent girls in Japan, an association that has not been clearly found in other developed countries.

Family income is important in controlling children’s BMI because healthier food tends to be expensive and participation in physical activities can be costly [26–29]. Previous studies have shown that childhood poverty induces breakfast skipping [30], which can lead to high BMI among adolescent girls who regularly skip breakfast [31, 32]. Our result for girls was similar to a previous study’s finding of an association between overweight and breakfast skipping at least once per week [33]. Moreover, breakfast skipping in adolescence is associated not only with overweight but also with larger waist circumference and higher levels of fasting insulin, total cholesterol, and low-density lipoprotein cholesterol [34]. Breakfast skipping is also associated with smoking, drinking, and physical inactivity [35]. Education programs that emphasize eating breakfast daily and target poor families with adolescent girls might serve to prevent childhood overweight and obesity in Japan. School lunch programs in Japan could also ensure a certain level of nutrition, and provide an opportunity to educate students on the importance of a healthy diet. Thus, improvements in school lunch programs and initiatives targeting breakfast skipping in poor families might be effective interventions in Japan, as they have been in the United States [36].

For girls, the association between economic status and BMI remained significant after adjusting for family structure. This finding only among girls is consistent with that of a previous study [37]. Childhood poverty and single-parent families need to be considered as different pathways leading to increased BMI. This is an important consideration because subsidies for single parents might not be effective for preventing overweight among girls. Further study is needed to elucidate the effect of poverty and single parenthood on weight.

This study has several limitations. First, due to its cross-sectional study design, reverse causality might explain the association between overweight adolescent girls and low economic status. Second, height, weight, economic status, and health status were self-reported. Specifically, self-reported BMI is reported to be slightly lower than measured BMI in adolescent girls [19]. However, on average, self-reported weight and BMI were found to be valid representations of their measured counterparts [38]. Third, although the response rate was high, not every student responded to the questionnaire. It is possible that students with lower economic status were less likely to respond to the questionnaire, so the strength of association might have been underestimated. However, the rate of students who considered their family’s financial situation to be “difficult” (boys, 4.28 %; girls, 3.35 %) was close to the rate of low household income reported in a previous study (5.8 %) [39]. Despite these limitations, due to a large sample from two communities, a substantial percentage of students with low economic status were identified. Further longitudinal studies are needed to confirm the association.

## Conclusion

This study clarified that low economic status mediated by breakfast skipping was significantly associated with higher BMI *z*-score in adolescent girls in Japan. Health policies at junior high schools to discourage breakfast skipping might be effective for countering the association between childhood poverty and overweight in adolescent girls.

## Additional file

Additional file 1: Questionnaire form in English. (DOCX 59 kb)

## Abbreviation

BMI: Body mass index
