# Supplementary material for: Association between economic status and body mass index among adolescents: a community-based cross-sectional study in Japan
Source: BMC Obes. 2016 Nov 10;3:47. doi: 10.1186/s40608-016-0127-z (PMC5103506; doi:10.1186/s40608-016-0127-z)
Supplement: Additional file 1: — Questionnaire form in English. (DOCX 59 kb) [file 40608_2016_127_MOESM1_ESM.docx]

**Health survey of student**

**Student** Hamamatsu University School of Medicine

- Please choose response options most accurately describing your views, and enter a circle or

write number in ( ) .

Q1．Age　　（　　　　　　　） years old

Q2．Sex　　1． Male 　・　 2．Female

【Please let me know about you】

Q3．How many days do you eat breakfast in a week?

Every day　 　5～6 days 　 3～4 days　 1～2 days　　Not eat

Q4．Usually, what time do you rise?　　　 Morning (　　　   ) o'clock　　(　　　   ) minutes

Q5.　Usually, what time do you go to bed?　Evening (　　　   ) o'clock　　(　　　   ) minutes

Q6．Weekday, How long time do you study in your home or cram school expect in school?

1. I study.

2. I do not study.

Mean of a daily

Abbreviation ( hours minutes)

Q7．How do you feel your grade attainment?

1. Satisfied 2. A little satisfied 3. Not much satisfied 4.Not satisfied

Q8．How do you feel your present healthy state？

1. Very good 2. Good 3. Usually 4. Not so good 5. Not good

Q9．What centimeter is the current your height?


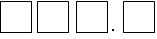


Ｃｍ


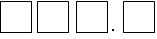
Q10．What kilograms current your weight?

Ｋｇ

Q11．How often do you eat meals from convenience stores or fast-food outlets?

1. Never 2. Some few a year 3. Some few a month 4. Once or two a week 4. Three or more week

Q12．Have you ever smoke even once until now?

1．No　　　　2．Yes

Q13.Are there any smoker in your family members?

1．No　 　2．Yes

（ Father ・ Mother ・ Other　）

**【**I hear about your household.】

Q14. Please enter circles to your household who live together.

1．Biological father　 　2．Biological mother　 3．Step father 　　　4．Step mother

5．Brother (Number: 　　 ) 　6． Grand-father 7．Grand-mother 8．Other

Q15．How do you feel your family’s economic affluence?

1. Not Affluent　 2．Not much Affluent 　3.Moderately　 4．Slightly affluent 　 5.Affluent
